# Exosomal tRNA-derived small RNA as a promising biomarker for cancer diagnosis

**DOI:** 10.1186/s12943-019-1000-8

**Published:** 2019-04-02

**Authors:** Lei Zhu, Jiao Li, Youling Gong, Qingbin Wu, Shuangyan Tan, Dan Sun, Xiaomin Xu, Yuanli Zuo, Yun Zhao, Yu-Quan Wei, Xia-Wei Wei, Yong Peng

**Affiliations:** 1 State Key Laboratory of Biotherapy and Cancer Center, National Clinical Research Center for Geriatrics, West China Hospital, Sichuan University, Chengdu, 610041 China; 2 Department of Thoracic Oncology, Cancer Center, West China Hospital, Sichuan University, Chengdu, 610041 China; 3 Department of Gastrointestinal Surgery, West China Hospital, Sichuan University, Chengdu, 610041 China; 4 Key Laboratory of Bio-Resource and Eco-Environment of Ministry of Education, College of Life Sciences, Sichuan University, Chengdu, 610064 China

**Keywords:** Exosome, Non-coding RNA, tRNA-derived small RNA, Plasma, Biomarker

## Abstract

tRNA-derived small RNA (tsRNA) is a novel regulatory small non-coding RNA and participates in diverse physiological and pathological processes. However, the presence of tsRNAs in exosome and their diagnostic potential remain unclear. In this study, we took advantage of small RNA-seq technology to profile exosomal tsRNAs from cell culture medium and plasma, and found ubiquitous presence of tsRNAs in exosome. To explore the potential value of tsRNA for cancer diagnosis, we compared exosomal tsRNA levels between liver cancer patients and healthy donors, revealing that tsRNAs were dramatically increased in plasma exosomes of liver cancer patients. Importantly, patients with liver cancer exhibited significantly higher levels of four tsRNAs (tRNA-ValTAC-3, tRNA-GlyTCC-5, tRNA-ValAAC-5 and tRNA-GluCTC-5) in plasma exosome, demonstrating that plasma exosomal tsRNA could serve as a novel diagnostic biomarker. Taken together, our results not only expand non-coding RNA species in exosome, but also highlight the potential of tsRNAs as a promising biomarker for cancer diagnosis.

**Electronic supplementary material:**

The online version of this article (10.1186/s12943-019-1000-8) contains supplementary material, which is available to authorized users.

## Main text

tRNA-derived small RNAs (tsRNAs), usually 18~40 nucleotides (nt) in length, are novel small non-coding RNAs (sncRNAs) generated from precursor or mature tRNAs [1]. tsRNAs can be grouped into three distinctive classes, including precursor tRNA-derived small RNAs with the characteristic of poly U residues at 3′ terminus (3′ U tRFs), and mature tRNA-derived fragments (tRFs) as well as halves (tRHs). And tRFs are further classified into 3 sub-groups: 5′ tRF, 3′ tRF and inter tRF [1]. Biogenesis of different tsRNAs is regulated by distinct mechanisms. For example, 3′ U tRFs are generated by RNase Z during tRNA maturation, while tRHs are derived from angiogenin cleavage within anti-codon loop of mature tRNAs [1, 2]. Increasing evidences indicate that tsRNAs expression is spatially and temporally controlled under physiological conditions, thus playing an important role in many biological processes [3–5]. For instance, sperm tsRNAs were discovered to be a paternal epigenetic factor mediating intergenerational inheritance of diet-induced metabolic disorders [4]. Recently, Croce’s group found that tsRNA expression is dysregulated in lung cancer and chronic lymphocytic leukemia, suggesting that tsRNAs participate in tumor initiation and development [6, 7].

Exosomes are membrane-bound vehicles with 30–100 nm in diameter secreted by most cell types and present in various types of body fluids, including plasma/serum, urine and saliva [8]. The exosome cargo consists of specific lipids, proteins and RNA molecules. Among them, microRNA (miRNA), circular RNA and long non-coding RNA have shown great potential as diagnostic or prognostic biomarkers [9, 10]. However, exosomal tsRNAs as diagnostic biomarkers have not been reported. In this study, we demonstrate the presence and expression pattern of tsRNAs in exosomes from cell culture media and patients’ plasma, highlighting their potential for cancer diagnosis.

## Results and discussion

### Profiling and validation of exosomal tsRNAs in cell culture media

**Fig. 1:**
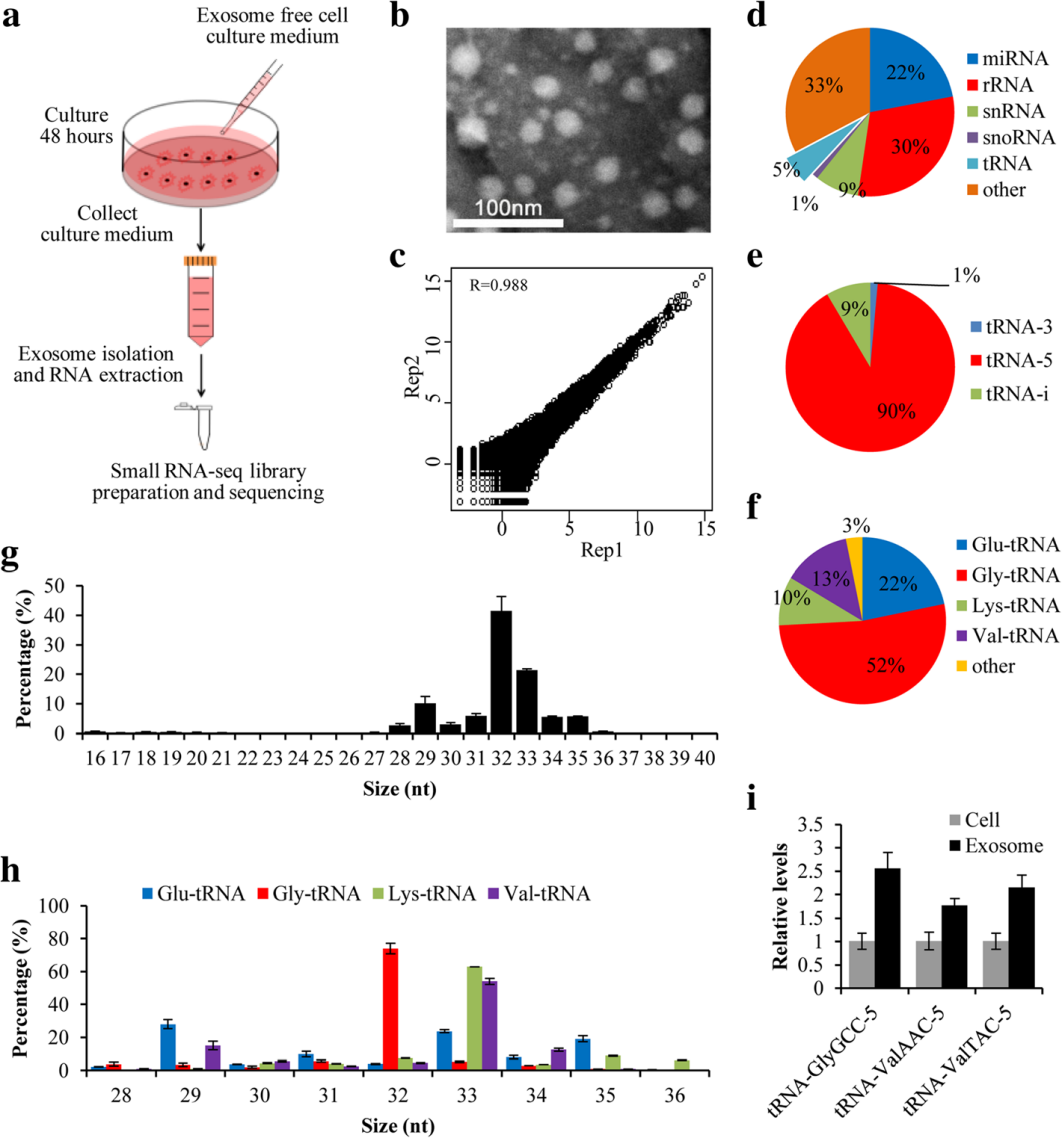
Profiling of tsRNAs in exosomes from cell culture medium. **a** Exosome collection procedure from SK-Hep1 cell culture medium; **b** Transmission electron micrograph (TEM) of exosomes isolated from cell culture medium (The scale bar is 100 nm); **c** Correlation analysis of two small RNA-seq replicates; **d** Percentage of each type of small RNAs in exosomes; **e** Percentage of each type of tsRNAs in exosomes; **f** Percentage of tRNA-5 generated from Glu-, Gly-, Lys- and Val-tRNA; **g** Length distribution of total tRNA-5s; **h** Length distribution of the tRNA-5s derived from Glu-, Gly-, Lys- and Val-tRNA; **i** Relative quantification of tsRNAs in SK-Hep1 cells and exosomes from cell culture medium

To comprehensively profile exosomal tsRNAs, we isolated exosomes from cultured medium of SK-Hep1 liver cancer cells and prepared small RNA-seq library for high-throughput sequencing (Fig. 1a). The prepared exosomes were confirmed by transmission electron microscopy (TEM), Western blot analysis (exosomal protein marker CD63 and non-exosomal marker Calnexin), and the particle size (30–100 nm) (Fig. 1b, Additional file 1: Figure S1, Additional file 2). The raw sequencing data were first cleaned through removing both 5′ and 3′ adaptors, and only high quality reads with 16–40 nt insertion were mapped to human genome and annotated (Additional file 2). We have performed exosomal small RNA sequencing twice with high reproducibility (R = 0.988; Fig. 1c). Our results showed that 5% of exosomal small RNAs were generated from tRNAs, suggesting that tsRNAs could be incorporated into exosomes and secreted into extracellular environment (Fig. 1d). Because tsRNAs generated from precursor tRNA have low levels in exosomes, so we focus on mature tRNA-derived tsRNAs that were further classified into tRNA-5 (5-termial of mature tRNA, 5′ tRF and 5′ tRH), tRF-i (internal of mature tRNA, i′ tRF) and tRNA-3 (3-termial of mature tRNA, 3′ tRF and 3′ tRH) according to their position (Additional file 1: Figure S2). As shown in Fig. 1e, tRNA-5 is the most abundant tsRNA in exosomes, accounting for 90%, whereas tRNA-3 and tRNA-i are 9 and 1%, respectively. We also analyzed the length distribution of tsRNAs and found that distributions of each type of tsRNA exhibited significant bias. Specifically, large majority of tRNA-5s were 32~33 nt in length (Fig. 1g), while tRNA-3s were mainly 16–18 nt in length and tRNA-i showed two peaks (16–17 nt and 28–32 nt) (Additional file 1: Figure S3).

We analyzed the origin of these exosomal tsRNAs and found that 97% of tRNA-5s came from four tRNAs (Gly-, Glu-, Lys- and Val-tRNA) (Fig. 1f). Moreover, tRNA-5s from Gly-tRNA were mainly 32 nt in length, while the majority of tRNA-5s from both Lys- and Val-tRNA were 33 nt in length (Fig. 1h), indicating that tsRNAs are not random degradation products, but generated by unknown mechanism. In addition, 52% of tRNA-5 and 32% of RNA-is were mainly derived from Gly-tRNA (Fig. 1f, Additional file 1: Figure S3D), whereas tRNA-3s were almost equally contributed by four different tRNAs (Ala-, Asp-, Glu- and Val-tRNA) (Additional file 1: Figure S3C). To validate these results, we performed quantitative PCR after reverse transcription (RT) of cellular and exosomal RNAs, and confirmed the existence of tsRNAs in exosomes (Fig. 1i and Additional file 3: Table S1, Additional file 2), suggesting that cells could sort cellular tsRNAs into exosomes.

### Identification of differentially expressed tsRNAs in liver cancer patients

**Fig. 2:**
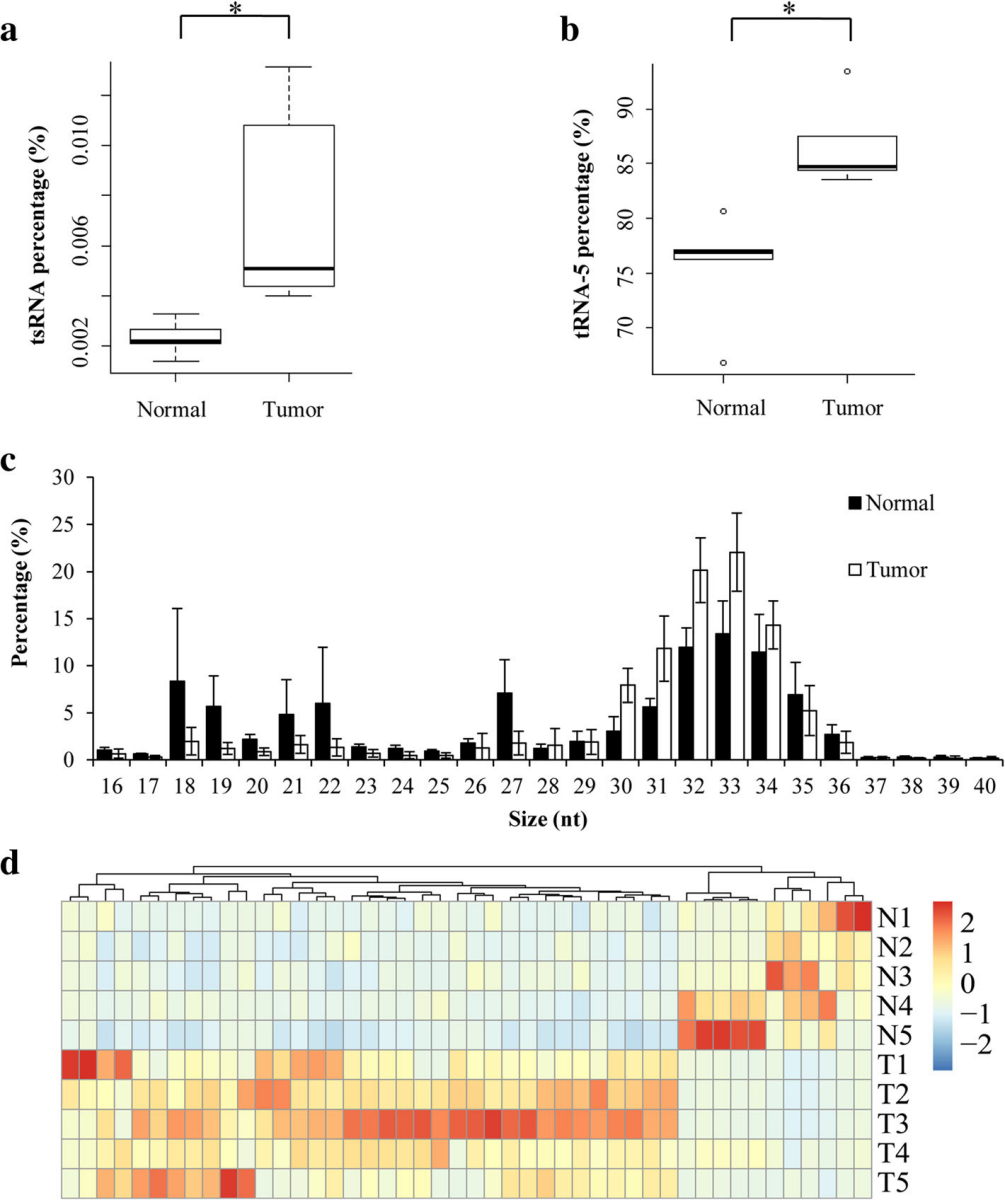
Profiling of tsRNAs in exosomes from plasma. **a**, **b** Relative expression of tsRNAs (**a**) and tRNA-5 (**b**) in exosomes from liver cancer patients and healthy donors; **c** Length distribution of tRNA-5 in exosomes from plasma; **d** Heatmap of differentially expressed tsRNAs between liver cancer patients and healthy donors. N1-N5: five healthy controls; T1-T5: five liver cancer patients

To explore the potential value of exosomal tsRNA for cancer diagnosis, we extracted and sequenced small RNAs in plasma exosomes from liver cancer patients and healthy donors (Additional file 2). Data analysis showed that miRNA was the most abundant small RNA species within plasma exsomes (Additional file 1: FigureS4A), supporting the role of miRNA as a potential biomarker. Interestingly, tsRNAs still account for 0.2–2% of total small RNAs in plasma exosomes (Additional file 1: Figure S4A). Moreover, tsRNA level in plasma exosomes from liver cancer patients was significantly increased when compared with that from healthy donors (Fig. 2a). Consistent with previous results from cell culture medium, tRNA-5 was also the major type of tsRNA in plasma exosomes (Additional file 1: Figure S4B). Furthermore, significantly higher level of tRNA-5 was observed in plasma exosomes from liver cancer patients than that in healthy controls (Fig. 2b), suggesting abnormal secretion of specific tsRNA into plasma exosomes of cancer patients.

Similar to the results from cell culture medium, the majority of tRNA-5s ranged from 31 nt to 34 nt in length (Fig. 2c) and tRNA-3s showed a peak at 18 nt (Additional file 1: Figure S5). Intriguingly, the level of tRNA-5s with 30~33 nt in length significantly increased in liver cancer patients (Fig. 2c), indicating that 30~33 nt tRNA-5s could be a potential biomarker for cancer diagnosis. In addition, most tRNA-5s in plasma exosomes were derived from Gly-tRNA, while the richest tRNA-3 was generated from Leu-tRNA (Additional file 1: Figure S6).

**Fig. 3:**
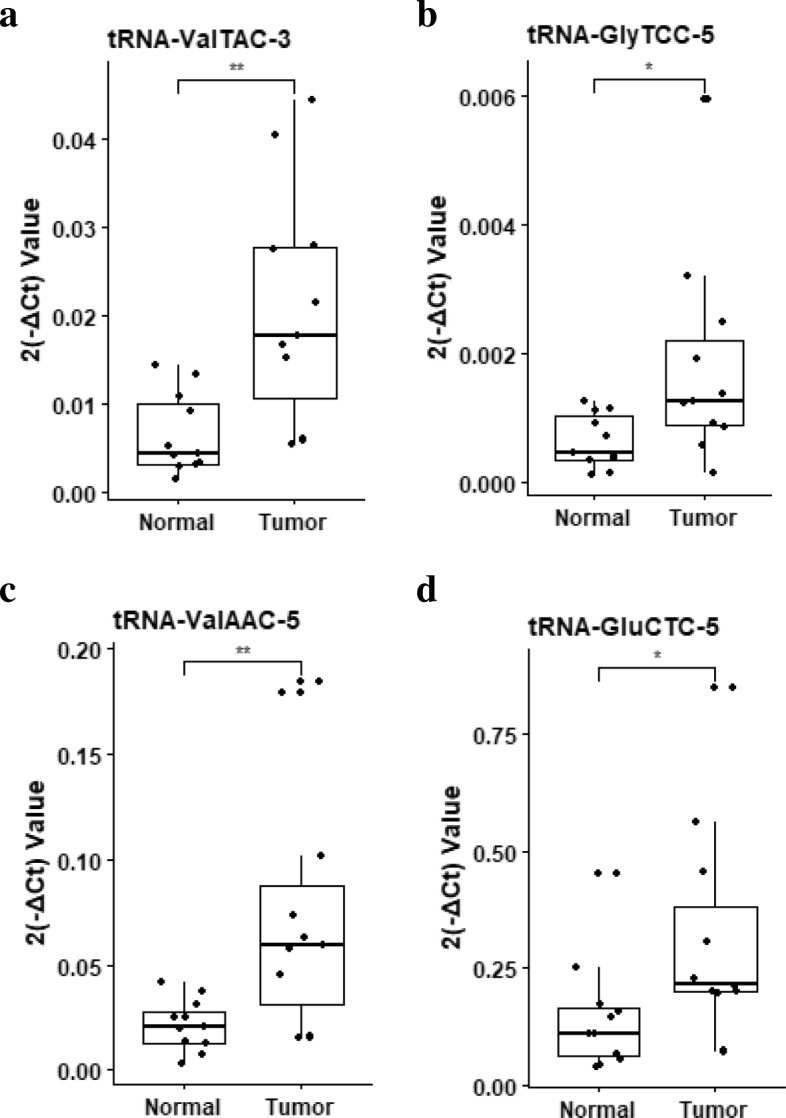
Validation of plasma exosomal tsRNAs in liver cancer patients and healthy controls. **a-d** RT-qPCR analyses of exosomal tRNA-ValTAC-3 (**a**), tRNA-GlyTCC-5 (**b**), tRNA-ValAAC-5 (**c**) and tRNA-GluCTC-5 (**d**) between healthy controls and liver cancer patients. miR-16 was used for normalization. *P* value of Student’s t-test: *p ≤ 0.05, **p ≤ 0.01

To identify the potential of tsRNAs for biomarker, we compared tsRNA levels in plasma exosomes and found that 46 tsRNAs (35 up-regulated, 11 down-regulated) were differentially expressed between liver cancer patients and healthy donors (Fig. 2d, Additional file 3: Table S2). Moreover, the RNA-seq results were validated individually by RT-qPCR analyses. Compared with healthy donors, the liver cancer patients exhibited significantly higher levels of tRNA-ValTAC-3, tRNA-GlyTCC-5, tRNA-ValAAC-5 and tRNA-GluCTC-5 in the plasma exosomes (Fig. 3, Additional file 3: Table S3), demonstrating that plasma exosomal tsRNA could serve as a novel biomarker for cancer diagnosis. However, more independent patient samples are needed to further validate the relationship between tsRNA and liver cancer.

## Conclusion

In this study, we demonstrated the existence of abundant tsRNA in exosomes from cell culture medium and plasma, representing a novel small RNA species in exosomes. Moreover, the plasma exosomes in liver cancer patients have significantly higher tsRNA level than that in healthy control. Notably, four tsRNAs from plasma exosomes are differentially expressed between liver cancer patients and healthy donors, indicating their great potential as a novel “liquid biopsy” biomarker for cancer diagnosis. Taken together, our study not only expands non-coding RNA species in exosome, but also sheds light on the diagnostic value of tsRNA as a promising biomarker for cancer.

## Additional files

Additional file 1: **Figure S1.** Identification of exosome isolated from cell culture medium. **Figure S2.** Classification of tsRNAs generated from mature tRNA. **Figure S3.** Length distribution and classification of tRNA-3 and tRNA-i in exosome from cell culture medium. **Figure S4.** Percentage of each RNA in plasma exosome. **Figure S5.** Length distribution of tRNA-3 and tRNA-i in plasma exosome from normal people and liver cancer patients. **Figure S6.** Classification of tRNA-5, tRNA-3 and tRNA-i from plasma exosome. (PPTX 1164 kb)

Additional file 2: Materials and Methods. (DOCX 43 kb)

Additional file 3: **Table S1.** List of tsRNA in exosome from cell culture medium for RT-qPCR. **Table S2.** List of 46 differentially expressed tsRNAs in plasma exosome between liver cancer patients and healthy donors. **Table S3.** List of differentially expressed tsRNA in patients for RT-qPCR. **Table S4.** Primers for reverse transcription and quantitative PCR. (DOCX 41 kb)

## Abbreviations

miRNA: microRNA
ncRNA: Non-coding RNA
RT-PCR: Reverse transcription polymerase chain reaction
TEM: Transmission electron microscopy
tsRNA: tRNA-derived small RNA
