# Supplementary material for: Exosomal tRNA-derived small RNA as a promising biomarker for cancer diagnosis
Source: Mol Cancer. 2019 Apr 2;18:74. doi: 10.1186/s12943-019-1000-8 (PMC6444574; doi:10.1186/s12943-019-1000-8)
Supplement: Supplementary file 1 — Figure S1. Identification of exosome isolated from cell culture medium. Figure S2. Classification of tsRNAs generated from mature tRNA. Figure S3. Length distribution and classification of tRNA-3 and tRNA-i in exosome from cell culture medium. Figure S4. Percentage of each RNA in plasma exosome. Figure S5. Length distribution of tRNA-3 and tRNA-i in plasma exosome from normal people and liver cancer patients. Figure S6. Classification of tRNA-5, tRNA-3 and tRNA-i from plasma exosome. (PPTX 1164 kb) [file 12943_2019_1000_MOESM1_ESM.pptx]

## Slide 1
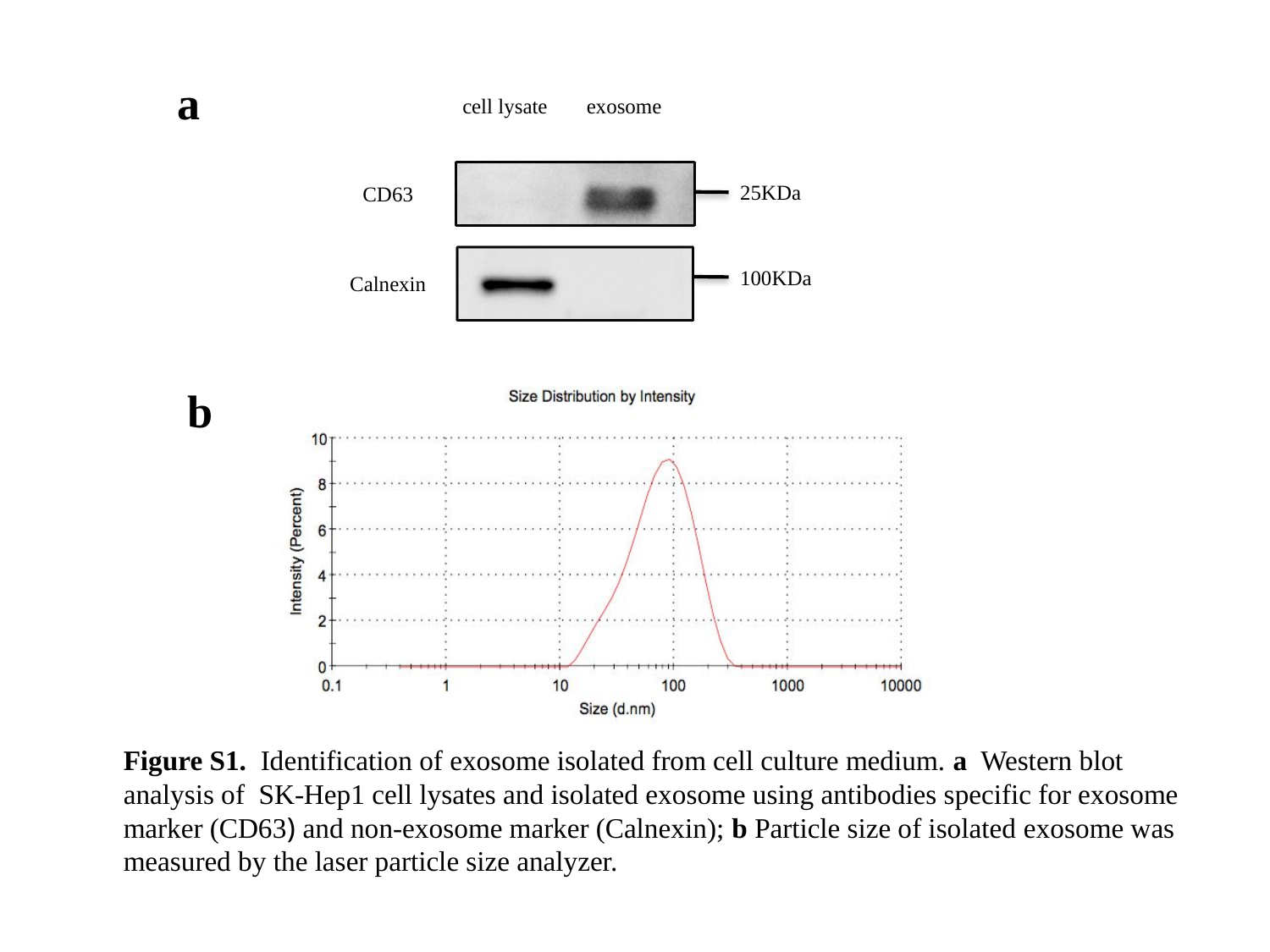

a
cell lysate
exosome
25KDa
CD63
100KDa
Calnexin
b
Figure S1. Identification of exosome isolated from cell culture medium. a Western blot analysis of SK-Hep1 cell lysates and isolated exosome using antibodies specific for exosome marker (CD63) and non-exosome marker (Calnexin); b Particle size of isolated exosome was measured by the laser particle size analyzer.

## Slide 2
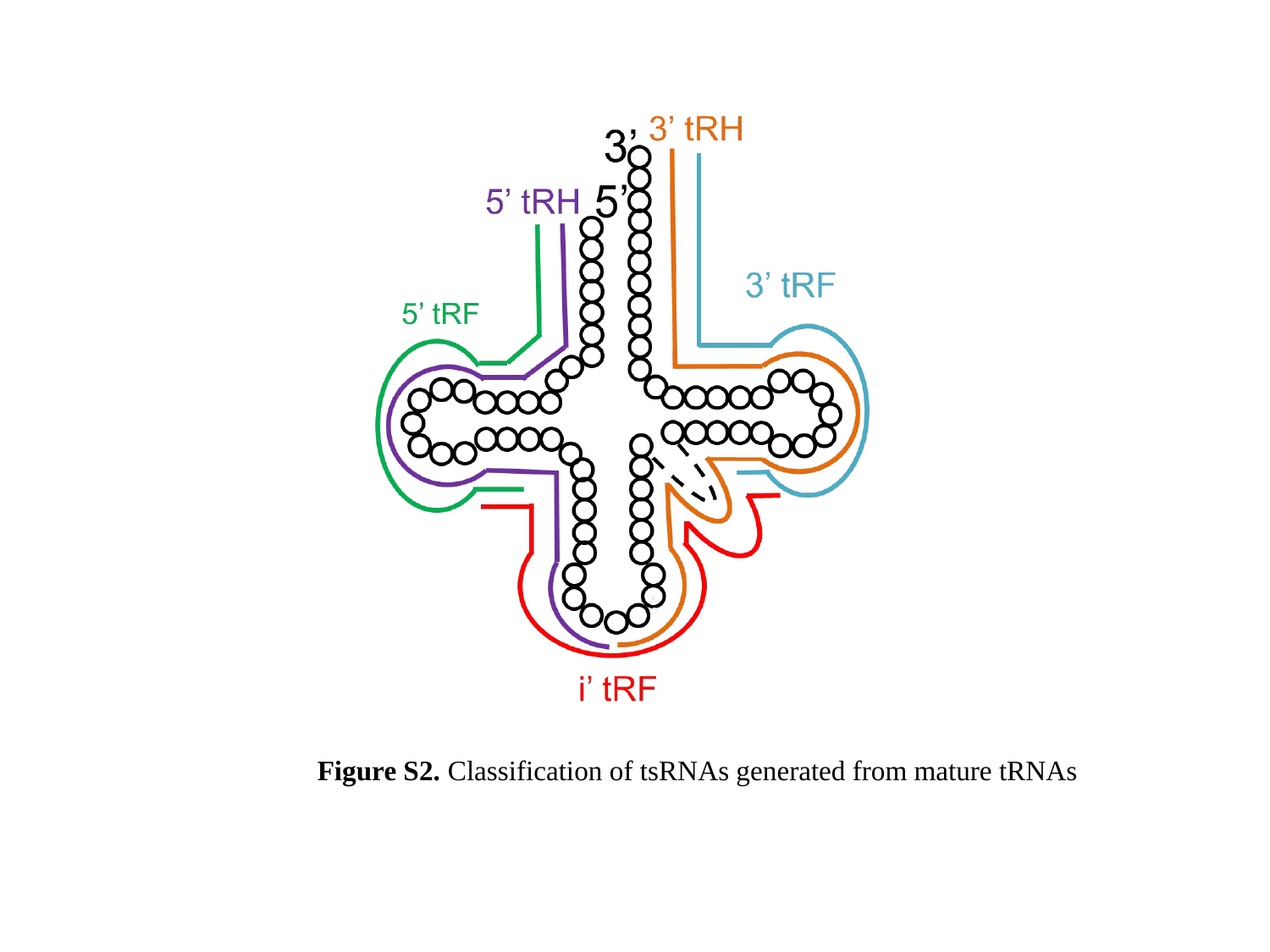

Figure S2. Classification of tsRNAs generated from mature tRNAs

## Slide 3
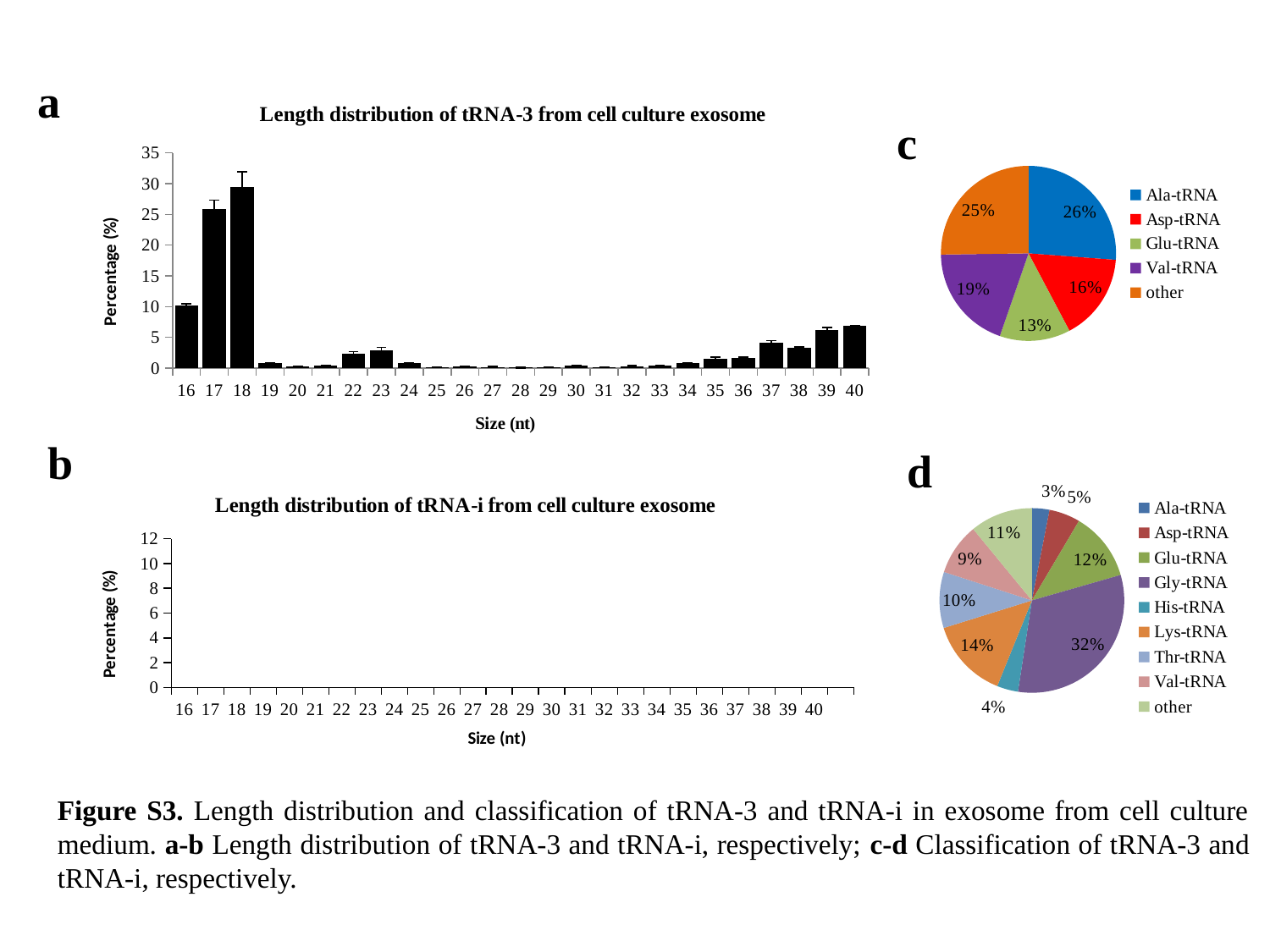

a
### Chart: Length distribution of tRNA-3 from cell culture exosome
| Category | tRNA-3 |
|---|---|
| 16 | 10.12734194991878 |
| 17 | 25.82855563442294 |
| 18 | 29.46204460554021 |
| 19 | 0.899452571837099 |
| 20 | 0.297151835065186 |
| 21 | 0.46519952855676 |
| 22 | 2.399717621460768 |
| 23 | 2.917175969044957 |
| 24 | 0.846449623317 |
| 25 | 0.114598267275873 |
| 26 | 0.213797704862803 |
| 27 | 0.205354160525661 |
| 28 | 0.0835029561009177 |
| 29 | 0.183000045484915 |
| 30 | 0.373401854017234 |
| 31 | 0.183297697281983 |
| 32 | 0.343497150030551 |
| 33 | 0.449503047070748 |
| 34 | 0.785151956358294 |
| 35 | 1.561562716582377 |
| 36 | 1.707256295033205 |
| 37 | 4.157337530719756 |
| 38 | 3.26186372626378 |
| 39 | 6.188227369138549 |
| 40 | 6.945558184089637 |c
### Chart
| Category | tRNA-3 |
|---|---|
| Ala-tRNA | 26.18865948993189 |
| Asp-tRNA | 16.08053068311848 |
| Glu-tRNA | 13.04258634514836 |
| Val-tRNA | 19.46714428445074 |
| other | 25.22107919735053 |b
d
### Chart
| Category | other |
|---|---|
| Ala-tRNA | 3.072971061313945 |
| Asp-tRNA | 5.43691534097261 |
| Glu-tRNA | 12.03943768792993 |
| Gly-tRNA | 31.85517453121889 |
| His-tRNA | 3.712628579262497 |
| Lys-tRNA | 14.06369441182336 |
| Thr-tRNA | 9.815542284330919 |
| Val-tRNA | 9.06513813414187 |
| other | 10.93849796900599 |
### Chart: Length distribution of tRNA-i from cell culture exosome
| Category | tRNA-i |
|---|---|
| 16 | 15.60404253615937 |
| 17 | 6.939818454145971 |
| 18 | 2.681788452272109 |
| 19 | 2.105087639098239 |
| 20 | 2.055542127925078 |
| 21 | 1.304405262361314 |
| 22 | 0.847320569963017 |
| 23 | 0.868947978184948 |
| 24 | 0.694762245998995 |
| 25 | 1.281925864550644 |
| 26 | 1.680857312593178 |
| 27 | 2.836375721231065 |
| 28 | 5.160996109676541 |
| 29 | 7.01069877560602 |
| 30 | 12.19068807554342 |
| 31 | 16.5946676262489 |
| 32 | 8.613065710790973 |
| 33 | 3.244277641528084 |
| 34 | 3.035220889240324 |
| 35 | 1.630371974536065 |
| 36 | 1.415349493336463 |
| 37 | 1.106800305480738 |
| 38 | 0.587451712010016 |
| 39 | 0.303549669191233 |
| 40 | 0.205987852327296 |Figure S3. Length distribution and classification of tRNA-3 and tRNA-i in exosome from cell culture medium. a-b Length distribution of tRNA-3 and tRNA-i, respectively; c-d Classification of tRNA-3 and tRNA-i, respectively.

## Slide 4
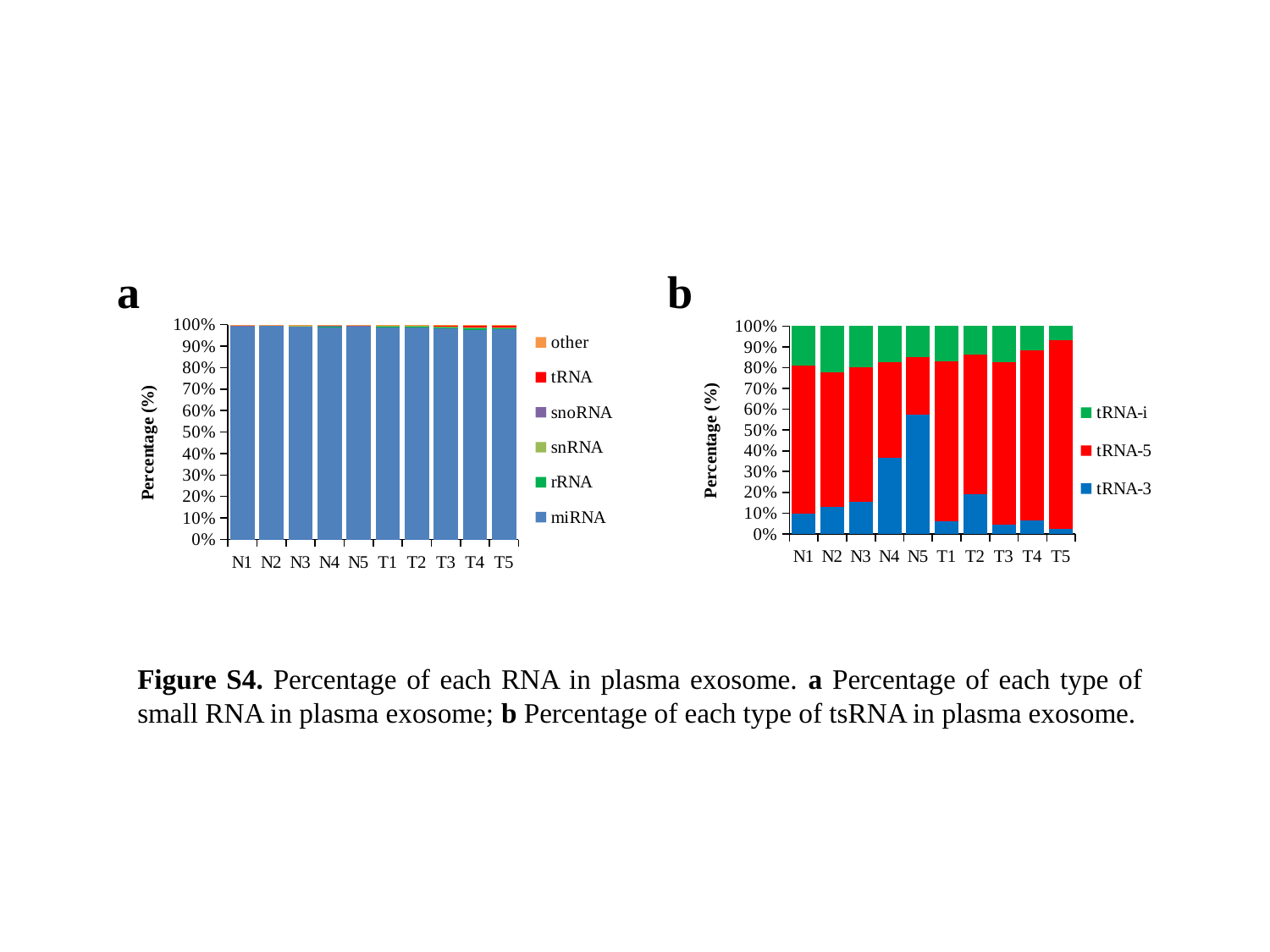

a
b
### Chart
| Category | miRNA | rRNA | snRNA | snoRNA | tRNA | other |
|---|---|---|---|---|---|---|
| N1 | 38136047.0 | 224514.0 | 10033.0 | 5504.0 | 84047.0 | 56905.0 |
| N2 | 41567628.0 | 135726.0 | 7417.0 | 1.0 | 58187.0 | 67108.0 |
| N3 | 41693882.0 | 246632.0 | 12716.0 | 7468.0 | 89561.0 | 67413.0 |
| N4 | 10887541.0 | 66624.0 | 2544.0 | 2277.0 | 36609.0 | 20960.0 |
| N5 | 28006058.0 | 75966.0 | 5096.0 | 9414.0 | 77046.0 | 43090.0 |
| T1 | 38337363.0 | 224179.0 | 11053.0 | 3.0 | 169763.0 | 57337.0 |
| T2 | 40698498.0 | 226890.0 | 14133.0 | 1.0 | 163127.0 | 65824.0 |
| T3 | 12689381.0 | 128887.0 | 8611.0 | 14.0 | 66284.0 | 32012.0 |
| T4 | 27370341.0 | 330703.0 | 13537.0 | 4.0 | 304262.0 | 73610.0 |
| T5 | 29845480.0 | 216089.0 | 12438.0 | 4.0 | 398647.0 | 57963.0 |
### Chart
| Category | tRNA-3 | tRNA-5 | tRNA-i |
|---|---|---|---|
| N1 | 7916.0 | 56965.0 | 15409.0 |
| N2 | 7617.0 | 37363.0 | 12934.0 |
| N3 | 13806.0 | 57552.0 | 17649.0 |
| N4 | 15616.0 | 19614.0 | 7469.0 |
| N5 | 56232.0 | 27216.0 | 14628.0 |
| T1 | 10386.0 | 127890.0 | 27960.0 |
| T2 | 33039.0 | 115304.0 | 23557.0 |
| T3 | 2902.0 | 49955.0 | 11055.0 |
| T4 | 17726.0 | 224783.0 | 31841.0 |
| T5 | 9262.0 | 349724.0 | 26111.0 |Figure S4. Percentage of each RNA in plasma exosome. a Percentage of each type of small RNA in plasma exosome; b Percentage of each type of tsRNA in plasma exosome.

## Slide 5
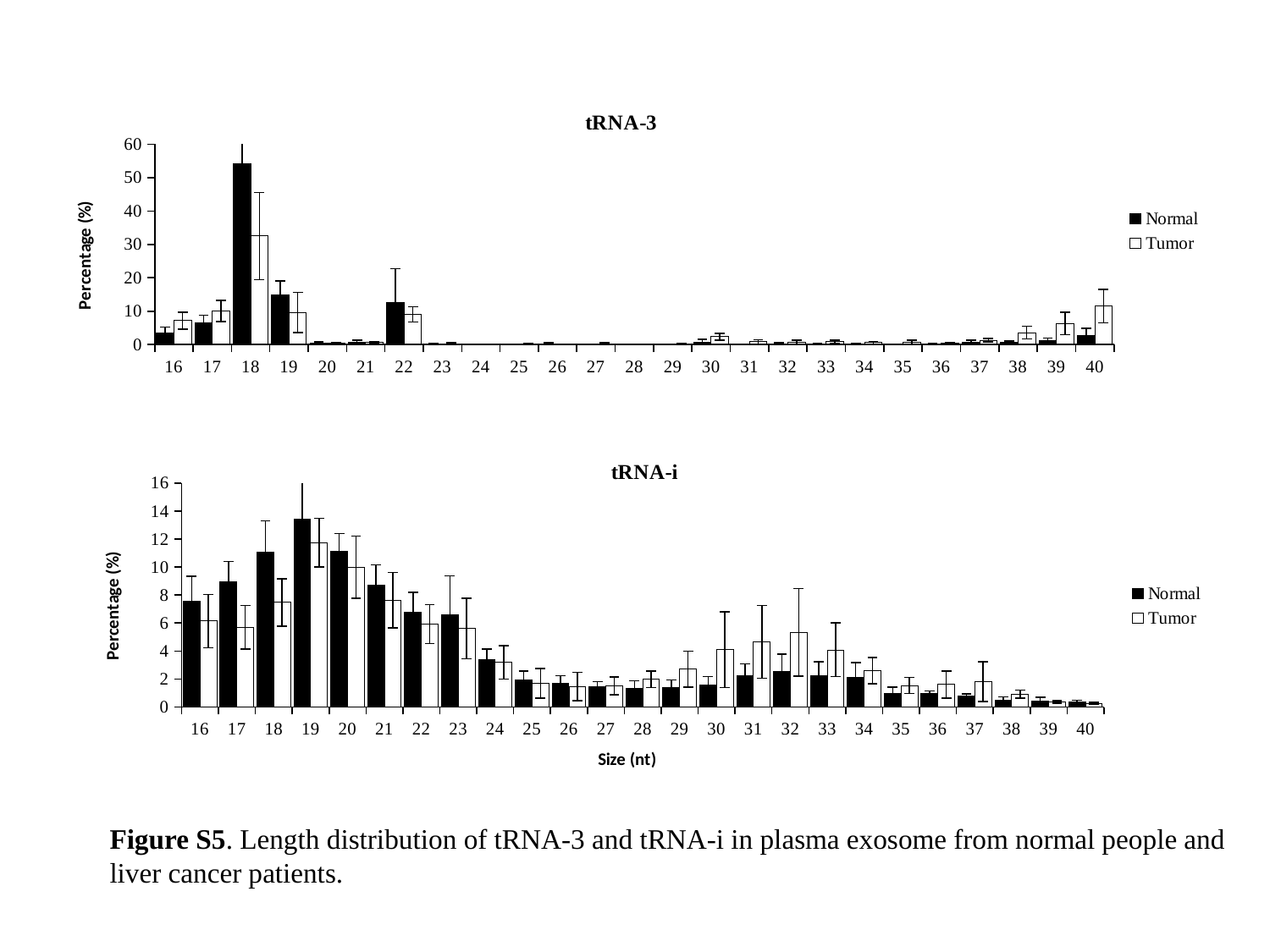

### Chart: tRNA-3
| Category | Normal | Tumor |
|---|---|---|
| 16 | 3.379053600521143 | 7.209260692687166 |
| 17 | 6.40144601863918 | 10.08291510108135 |
| 18 | 54.22750554159162 | 32.5128961808741 |
| 19 | 14.82778114373846 | 9.656836932600767 |
| 20 | 0.529584211843462 | 0.521773360508953 |
| 21 | 0.746396930565004 | 0.751160356119292 |
| 22 | 12.56485332050807 | 9.066298062115171 |
| 23 | 0.181955402837733 | 0.3091103852631 |
| 24 | 0.0739334596061597 | 0.122259198788493 |
| 25 | 0.0328449492761518 | 0.116414489208845 |
| 26 | 0.21394141175354 | 0.0105665965144458 |
| 27 | 0.0168604291950341 | 0.273606396249324 |
| 28 | 0.0115547597155326 | 0.00286191746482606 |
| 29 | 0.0597683475245957 | 0.2019009073214 |
| 30 | 0.786458374526176 | 2.341084896520374 |
| 31 | 0.0771486098167305 | 0.811597450563048 |
| 32 | 0.208005776509413 | 0.752171696878716 |
| 33 | 0.297234691185013 | 0.851833126478578 |
| 34 | 0.153410243755417 | 0.573347739894268 |
| 35 | 0.119419109476141 | 0.637670578199541 |
| 36 | 0.126671716500646 | 0.421148763515506 |
| 37 | 0.60386102671503 | 1.303432716856657 |
| 38 | 0.593668669734629 | 3.601442204664589 |
| 39 | 1.112032465395573 | 6.323230366619198 |
| 40 | 2.654609789069542 | 11.5451798830123 |
### Chart: tRNA-i
| Category | Normal | Tumor |
|---|---|---|
| 16 | 7.570334575955963 | 6.136040766194316 |
| 17 | 8.94888445122017 | 5.700334064022378 |
| 18 | 11.05823394174 | 7.471304412803491 |
| 19 | 13.40229153624197 | 11.75024838293465 |
| 20 | 11.10413927583321 | 9.99952426040862 |
| 21 | 8.695848679880235 | 7.625208865827014 |
| 22 | 6.762411711145924 | 5.917215886896044 |
| 23 | 6.573336406759415 | 5.607462337828787 |
| 24 | 3.402720472186387 | 3.192475736882971 |
| 25 | 1.951515873977431 | 1.689022617413049 |
| 26 | 1.682977399177638 | 1.466112665615215 |
| 27 | 1.443375197350511 | 1.51340281591165 |
| 28 | 1.344582439980299 | 1.973035181378433 |
| 29 | 1.407810558962458 | 2.705436746414663 |
| 30 | 1.57599793940476 | 4.093502502289654 |
| 31 | 2.234307428564885 | 4.666013833890139 |
| 32 | 2.552581358064293 | 5.326526976547253 |
| 33 | 2.237033461291721 | 4.078786637178831 |
| 34 | 2.136690847312758 | 2.602929068149886 |
| 35 | 0.938024445618735 | 1.528685628183136 |
| 36 | 0.936553678300372 | 1.60093185034672 |
| 37 | 0.779888857178026 | 1.811114145069171 |
| 38 | 0.482796987971411 | 0.918732657155337 |
| 39 | 0.433710800529395 | 0.358805926993002 |
| 40 | 0.343951675352032 | 0.267146033665579 |Figure S5. Length distribution of tRNA-3 and tRNA-i in plasma exosome from normal people and liver cancer patients.

## Slide 6
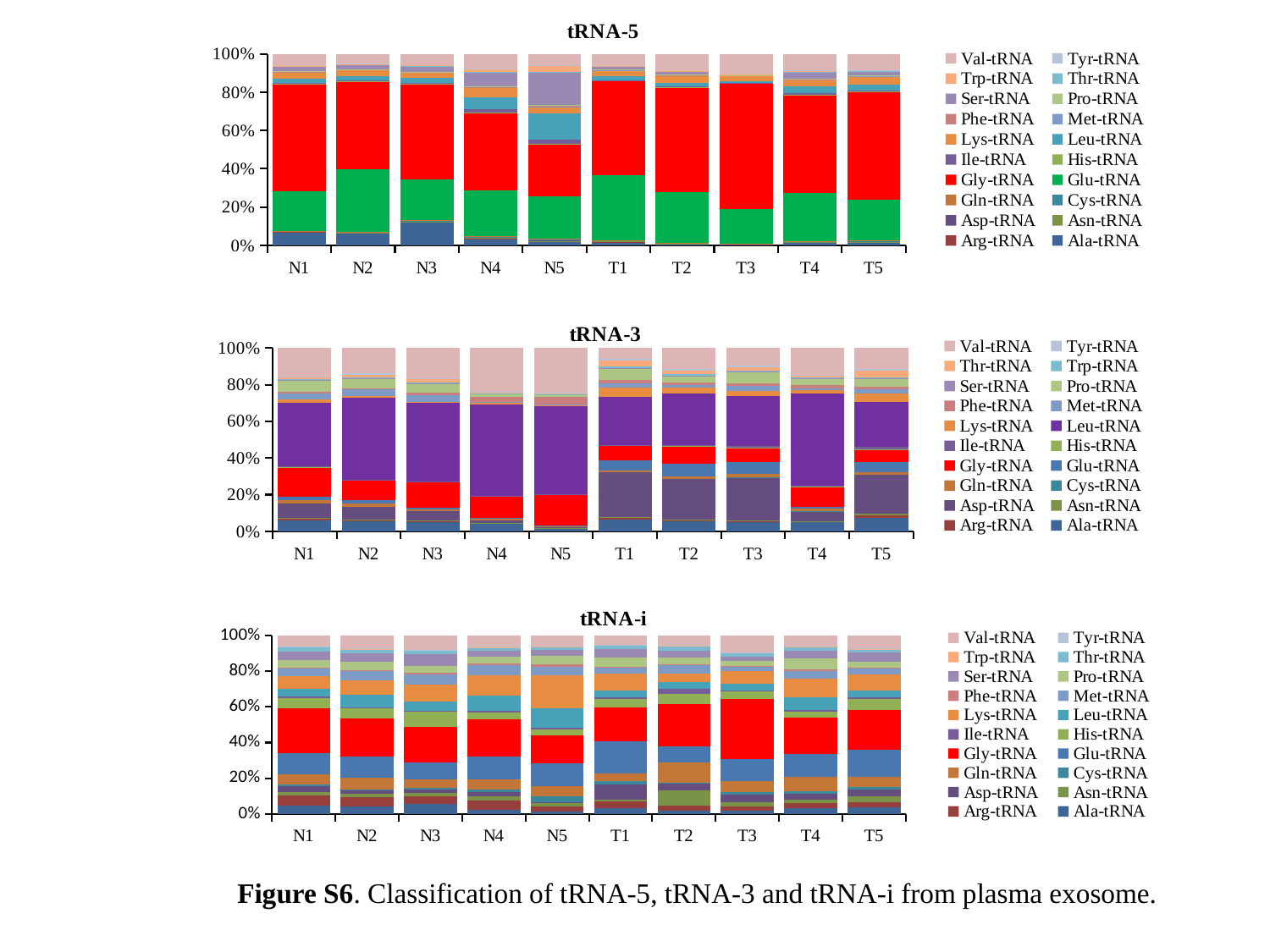

### Chart: tRNA-5
| Category | Ala-tRNA | Arg-tRNA | Asn-tRNA | Asp-tRNA | Cys-tRNA | Gln-tRNA | Glu-tRNA | Gly-tRNA | His-tRNA | Ile-tRNA | Leu-tRNA | Lys-tRNA | Met-tRNA | Phe-tRNA | Pro-tRNA | Ser-tRNA | Thr-tRNA | Trp-tRNA | Tyr-tRNA | Val-tRNA |
|---|---|---|---|---|---|---|---|---|---|---|---|---|---|---|---|---|---|---|---|---|
| N1 | 3994.0 | 119.0 | 25.0 | 123.0 | 28.0 | 32.0 | 11700.0 | 31935.0 | 66.0 | 256.0 | 1246.0 | 1899.0 | 243.0 | 23.0 | 136.0 | 1474.0 | 21.0 | 156.0 | 8.0 | 3473.0 |
| N2 | 2322.0 | 104.0 | 49.0 | 156.0 | 25.0 | 21.0 | 12196.0 | 17043.0 | 59.0 | 286.0 | 743.0 | 1175.0 | 130.0 | 17.0 | 30.0 | 967.0 | 12.0 | 59.0 | 13.0 | 1945.0 |
| N3 | 6992.0 | 133.0 | 28.0 | 209.0 | 80.0 | 43.0 | 12442.0 | 28444.0 | 115.0 | 321.0 | 1556.0 | 1594.0 | 115.0 | 22.0 | 47.0 | 1690.0 | 14.0 | 214.0 | 5.0 | 3446.0 |
| N4 | 624.0 | 107.0 | 39.0 | 113.0 | 16.0 | 30.0 | 4701.0 | 7988.0 | 22.0 | 315.0 | 1247.0 | 885.0 | 108.0 | 45.0 | 34.0 | 1452.0 | 72.0 | 298.0 | 28.0 | 1482.0 |
| N5 | 502.0 | 87.0 | 36.0 | 249.0 | 6.0 | 47.0 | 6057.0 | 7362.0 | 16.0 | 691.0 | 3667.0 | 930.0 | 163.0 | 60.0 | 48.0 | 4581.0 | 99.0 | 973.0 | 28.0 | 1605.0 |
| T1 | 3851.0 | 378.0 | 309.0 | 859.0 | 209.0 | 112.0 | 76300.0 | 111579.0 | 207.0 | 184.0 | 4610.0 | 6370.0 | 1102.0 | 13.0 | 261.0 | 3554.0 | 145.0 | 250.0 | 39.0 | 14389.0 |
| T2 | 774.0 | 155.0 | 73.0 | 419.0 | 191.0 | 85.0 | 34125.0 | 70024.0 | 79.0 | 186.0 | 2666.0 | 4168.0 | 836.0 | 19.0 | 92.0 | 2255.0 | 72.0 | 198.0 | 9.0 | 11444.0 |
| T3 | 1113.0 | 196.0 | 72.0 | 521.0 | 359.0 | 186.0 | 64329.0 | 230423.0 | 178.0 | 163.0 | 2978.0 | 8937.0 | 981.0 | 14.0 | 176.0 | 1904.0 | 33.0 | 86.0 | 20.0 | 37031.0 |
| T4 | 1696.0 | 207.0 | 85.0 | 380.0 | 120.0 | 62.0 | 28953.0 | 59168.0 | 67.0 | 849.0 | 4157.0 | 3554.0 | 722.0 | 29.0 | 163.0 | 4133.0 | 167.0 | 868.0 | 39.0 | 9847.0 |
| T5 | 749.0 | 88.0 | 75.0 | 142.0 | 50.0 | 90.0 | 10722.0 | 28284.0 | 57.0 | 163.0 | 1399.0 | 2165.0 | 283.0 | 5.0 | 33.0 | 1202.0 | 40.0 | 92.0 | 13.0 | 4273.0 |
### Chart: tRNA-3
| Category | Ala-tRNA | Arg-tRNA | Asn-tRNA | Asp-tRNA | Cys-tRNA | Gln-tRNA | Glu-tRNA | Gly-tRNA | His-tRNA | Ile-tRNA | Leu-tRNA | Lys-tRNA | Met-tRNA | Phe-tRNA | Pro-tRNA | Ser-tRNA | Trp-tRNA | Thr-tRNA | Tyr-tRNA | Val-tRNA |
|---|---|---|---|---|---|---|---|---|---|---|---|---|---|---|---|---|---|---|---|---|
| N1 | 482.0 | 61.0 | 24.0 | 643.0 | 0.0 | 148.0 | 149.0 | 1251.0 | 25.0 | 19.0 | 2747.0 | 160.0 | 249.0 | 85.0 | 473.0 | 5.0 | 53.0 | 94.0 | 30.0 | 1216.0 |
| N2 | 440.0 | 23.0 | 14.0 | 537.0 | 0.0 | 127.0 | 135.0 | 850.0 | 3.0 | 19.0 | 3391.0 | 97.0 | 253.0 | 63.0 | 402.0 | 2.0 | 50.0 | 102.0 | 56.0 | 1053.0 |
| N3 | 706.0 | 87.0 | 34.0 | 691.0 | 0.0 | 129.0 | 106.0 | 1954.0 | 15.0 | 45.0 | 5898.0 | 84.0 | 527.0 | 204.0 | 664.0 | 12.0 | 73.0 | 229.0 | 71.0 | 2277.0 |
| N4 | 677.0 | 32.0 | 8.0 | 210.0 | 0.0 | 113.0 | 75.0 | 1859.0 | 4.0 | 32.0 | 7822.0 | 125.0 | 72.0 | 430.0 | 236.0 | 18.0 | 33.0 | 60.0 | 74.0 | 3735.0 |
| N5 | 954.0 | 64.0 | 26.0 | 148.0 | 0.0 | 581.0 | 19.0 | 9530.0 | 38.0 | 131.0 | 27040.0 | 427.0 | 88.0 | 2120.0 | 758.0 | 117.0 | 39.0 | 123.0 | 313.0 | 13716.0 |
| T1 | 1115.0 | 201.0 | 72.0 | 4338.0 | 0.0 | 186.0 | 922.0 | 1399.0 | 56.0 | 54.0 | 4641.0 | 919.0 | 405.0 | 301.0 | 1187.0 | 6.0 | 170.0 | 517.0 | 150.0 | 1077.0 |
| T2 | 583.0 | 49.0 | 32.0 | 2297.0 | 0.0 | 161.0 | 721.0 | 939.0 | 30.0 | 59.0 | 2957.0 | 307.0 | 154.0 | 149.0 | 360.0 | 11.0 | 88.0 | 187.0 | 107.0 | 1190.0 |
| T3 | 443.0 | 85.0 | 21.0 | 2169.0 | 3.0 | 166.0 | 623.0 | 706.0 | 16.0 | 77.0 | 2531.0 | 266.0 | 235.0 | 142.0 | 576.0 | 2.0 | 70.0 | 173.0 | 67.0 | 889.0 |
| T4 | 1619.0 | 163.0 | 43.0 | 1777.0 | 5.0 | 422.0 | 410.0 | 3658.0 | 25.0 | 79.0 | 16724.0 | 528.0 | 247.0 | 735.0 | 1051.0 | 70.0 | 159.0 | 311.0 | 187.0 | 4826.0 |
| T5 | 208.0 | 44.0 | 21.0 | 617.0 | 0.0 | 43.0 | 160.0 | 195.0 | 10.0 | 34.0 | 715.0 | 141.0 | 55.0 | 45.0 | 131.0 | 4.0 | 17.0 | 108.0 | 26.0 | 326.0 |
### Chart: tRNA-i
| Category | Ala-tRNA | Arg-tRNA | Asn-tRNA | Asp-tRNA | Cys-tRNA | Gln-tRNA | Glu-tRNA | Gly-tRNA | His-tRNA | Ile-tRNA | Leu-tRNA | Lys-tRNA | Met-tRNA | Phe-tRNA | Pro-tRNA | Ser-tRNA | Thr-tRNA | Trp-tRNA | Tyr-tRNA | Val-tRNA |
|---|---|---|---|---|---|---|---|---|---|---|---|---|---|---|---|---|---|---|---|---|
| N1 | 692.0 | 867.0 | 347.0 | 490.0 | 142.0 | 874.0 | 1813.0 | 3889.0 | 877.0 | 165.0 | 631.0 | 1154.0 | 595.0 | 102.0 | 669.0 | 738.0 | 336.0 | 60.0 | 19.0 | 947.0 |
| N2 | 518.0 | 667.0 | 246.0 | 264.0 | 86.0 | 871.0 | 1518.0 | 2757.0 | 739.0 | 81.0 | 894.0 | 1033.0 | 701.0 | 72.0 | 578.0 | 613.0 | 270.0 | 95.0 | 48.0 | 883.0 |
| N3 | 965.0 | 739.0 | 328.0 | 349.0 | 182.0 | 847.0 | 1644.0 | 3524.0 | 1514.0 | 70.0 | 983.0 | 1683.0 | 1006.0 | 131.0 | 693.0 | 1178.0 | 295.0 | 60.0 | 71.0 | 1382.0 |
| N4 | 182.0 | 377.0 | 161.0 | 177.0 | 127.0 | 409.0 | 974.0 | 1545.0 | 292.0 | 65.0 | 661.0 | 837.0 | 433.0 | 77.0 | 285.0 | 226.0 | 122.0 | 36.0 | 32.0 | 451.0 |
| N5 | 160.0 | 450.0 | 265.0 | 111.0 | 425.0 | 865.0 | 1853.0 | 2311.0 | 507.0 | 108.0 | 1598.0 | 2742.0 | 682.0 | 172.0 | 738.0 | 494.0 | 175.0 | 69.0 | 89.0 | 811.0 |
| T1 | 1064.0 | 1102.0 | 363.0 | 2792.0 | 476.0 | 1376.0 | 5794.0 | 6013.0 | 1450.0 | 309.0 | 1294.0 | 2989.0 | 1045.0 | 151.0 | 1706.0 | 1437.0 | 706.0 | 53.0 | 64.0 | 1611.0 |
| T2 | 434.0 | 811.0 | 2426.0 | 1113.0 | 126.0 | 3140.0 | 2478.0 | 6653.0 | 1665.0 | 702.0 | 1065.0 | 1368.0 | 1355.0 | 151.0 | 1097.0 | 1013.0 | 679.0 | 145.0 | 81.0 | 1451.0 |
| T3 | 477.0 | 605.0 | 564.0 | 1194.0 | 338.0 | 1660.0 | 3181.0 | 8752.0 | 1196.0 | 154.0 | 943.0 | 1823.0 | 711.0 | 78.0 | 729.0 | 674.0 | 470.0 | 87.0 | 60.0 | 2415.0 |
| T4 | 763.0 | 681.0 | 440.0 | 741.0 | 367.0 | 1899.0 | 3067.0 | 4778.0 | 782.0 | 224.0 | 1687.0 | 2436.0 | 1002.0 | 207.0 | 1443.0 | 1021.0 | 491.0 | 146.0 | 74.0 | 1299.0 |
| T5 | 397.0 | 327.0 | 341.0 | 449.0 | 134.0 | 655.0 | 1678.0 | 2442.0 | 680.0 | 117.0 | 450.0 | 967.0 | 393.0 | 58.0 | 354.0 | 541.0 | 203.0 | 33.0 | 26.0 | 802.0 |Figure S6. Classification of tRNA-5, tRNA-3 and tRNA-i from plasma exosome.
