# Supplementary material for: Exosomal tRNA-derived small RNA as a promising biomarker for cancer diagnosis
Source: Mol Cancer. 2019 Apr 2;18:74. doi: 10.1186/s12943-019-1000-8 (PMC6444574; doi:10.1186/s12943-019-1000-8)
Supplement: Supplementary file 2 — Materials and Methods. (DOCX 43 kb) [file 12943_2019_1000_MOESM2_ESM.docx]

**Materials and Methods**

**Cell culture**

SK-Hep1 cells were cultured in Dulbecco’s modified Eagle’s medium (DMEM) supplemented with 10% exosome-free fetal bovine serum (FBS) and 1% penicillin-streptomycin at 37°C in 5% CO_2_ incubator. To remove exosome, FBS was subjected to ultracentrifugation at 170,000 x g for 14 hours at 4°C.

## Exosome isolation

To isolate cell-derived exosomes, cells were cultured in exosome-free medium for 48 h, and then the media were collected to prepare exosomes using Total Exosome Isolation Reagent (from cell culture media) (Cat#4478359, Invitrogen, USA) following the manufacturer’s protocol. Plasma samples were collected from West China Hospital (China), which was approved by the Ethics Committee of West China Hospital of Sichuan University. Written informed consent for research purposes was provided for the patients. For plasma samples, the exosomes were isolated using Total Exosome Isolation Kit (from plasma) (Cat#4484450, Invitrogen, USA) following the manufacturer’s protocol. The isolated exosomes were resuspended in PBS and used immediately or stored at –80°C.

**Nanoparticle analysis**

The size distribution of exosomes was measured by a laser particle size analyzer (Malvern Nano-ZS 90, UK) utilizing dynamic light scattering (DLS) technique. Samples were diluted using PBS and measured in triplicate.

**Transmission electron microscopy (TEM)**

30 µl of isolated exosomes was placed on a copper mesh and negatively stained with 2% phosphotungstic acid solution for 10 min. The sample was then dried for 2 min under incandescent light. The copper mesh was observed and photographed under a transmission electron microscope (H-7650 Hitachi microscope, Japan)

**Protein preparation and Western blot analysis**

Proteins were extracted from exosomes or collected cells using RIPA lysis buffer plus protease inhibitor cocktails, followed by sonication on ice and centrifugation at 12,000g for 15min at 4°C. After measurement by BCA protein assay kit (Beyotime, China), the proteins were subjected to SDS-PAGE and transfer onto PVDF membrane (Millipore). The membrane was blocked by 5% non-fat dry milk in TBST solution for 1 h at room temperature and then incubated with the primary antibody at 4°C overnight. After washed for 3 times with TBST solution, the membrane was incubated with horseradish peroxidase-conjugated secondary antibody at room temperature for 1 h. Finally, protein bands were detected using SuperSignal West Dura Extended Duration Substrate (Thermo Scientific, USA). The primary antibodies include anti-CD63 (Abcam, Cambridge, UK) and anti-Calnexin (Zen-Bioscience, Chengdu, China).

**RNA extraction and small RNA sequencing**

Total RNAs from exosomes were extracted using TRIzol LS Reagent (Invitrogen, USA). Small RNA sequencing library was prepared by NEXTflex Small RNA-seq Kit v3 (BIOO SCIENTIFIC, USA) following the manufacturer’s protocol and sequenced on Illumina X Ten sequencing platform (Novogene, China).

**Reverse transcription and quantitative real-time PCR (RT-qPCR)**

Total RNAs was subjected to cDNA synthesis by M-MLV Reverse Transcriptase (Invitrogen, USA), and qPCR was performed with SYBR Premix Ex Taq (Takara Bio, China) using StepOne Plus real-time PCR system (Applied Biosystems). Based on the reported literatures [1] ~~[2]~~, miR-16 was chosen as internal control for tsRNA quantification in plasma exosomes. The relative expression levels were calculated via the 2^-∆∆Ct^ method. The primers for RT and qPCR are listed in Table S4.

**Small RNA-seq analysis**

The raw sequencing data was cleaned through removing 5’ and 3’ adaptor and low quality reads were filtered out. The identical reads were merged and the number of each unique reads was counted. Only reads with 16-40 nt insertion were kept for further analysis. Mature tRNA genes and 100 bp downstream of tRNA genes were retrieved from UCSC (hg38). To annotate exosomal small RNAs, cleaned reads were mapped to human genome and classified them into miRNA, tRNA, rRNA, snRNA, snoRNA and others according to gene annotation of hg38. To identify tsRNAs, reads were first aligned to mature tRNAs and downstream sequences with blast [2]~~[1]~~. Only the reads perfectly matched to genome were counted and classified into 5’-, 3’-, i’- and 3’ U of tRNA according to the positions where tsRNAs are generated from. To allow quantitative comparisons, the expression level of tsRNA was normalized with total tsRNA (1,000,000 * tsRNA read count/total tsRNA count, RPM). Differentially expressed tsRNA was identified by R package, Deseq2.

**Statistical analysis**

The results are presented as the means ± S.D., and the data were subjected to Student’s t-test or Pearson’s correlation analysis.

**References**

1. Fong MY, Zhou W, Liu L, Alontaga AY, Chandra M, Ashby J. et al. Breast-cancer-secreted miR-122 reprograms glucose metabolism in premetastatic niche to promote metastasis. Nat Cell Biol. 2015. 17(2): p. 183-94.

2. Altschul SF, Gish W, Miller W, Myers EW, Lipman DJ. Basic local alignment search tool. J Mol Biol.1990. 215(3): p. 403-10.
