# Supplementary material for: Exosomal tRNA-derived small RNA as a promising biomarker for cancer diagnosis
Source: Mol Cancer. 2019 Apr 2;18:74. doi: 10.1186/s12943-019-1000-8 (PMC6444574; doi:10.1186/s12943-019-1000-8)
Supplement: Supplementary file 3 — Table S1. List of tsRNA in exosome from cell culture medium for RT-qPCR. Table S2. List of 46 differentially expressed tsRNAs in plasma exosome between liver cancer patients and healthy donors. Table S3. List of differentially expressed tsRNA in patients for RT-qPCR. Table S4. Primers for reverse transcription and quantitative PCR. (DOCX 41 kb) [file 12943_2019_1000_MOESM3_ESM.docx]

**Table S1. List of tsRNA in exosome from cell culture medium for RT-qPCR**

| tsRNA-ID | Sequence |
| --- | --- |
| tRNA-GlyGCC-5 | GCATGGGTGGTTCAGTGGTAGAATTCTCGCCT |
| tRNA-ValAAC-5 | GTTTCCGTAGTGTAGTGGTTATCACGTTCGCCT |
| tRNA-ValTAC-5 | GGTTCCATAGTGTAGTGGTTATCACGTCTGCTT |

**Table S2. List of 46 differentially expressed tsRNAs in plasma exosome between liver cancer patients and healthy donors**

| tsRNA sequence | log_2_FC | *P*_adj_ | N1 | N2 | N3 | N4 | N5 | T1 | T2 | T3 | T4 | T5 | tRNA gene | tsRNA type |
| --- | --- | --- | --- | --- | --- | --- | --- | --- | --- | --- | --- | --- | --- | --- |
| GCGTTGGTGGTATAGTGGTGAGCATAGCTGCCTT | 3.81 | 0.008 | 62 | 328 | 225 | 492 | 20 | 1480 | 2328 | 2402 | 1507 | 1533 | Gly-TCC | tRNA-5 |
| GCGTTGGTGGTATAGTGGTTAGCATAGCTGCCTT | 3.50 | 0.008 | 834 | 380 | 640 | 656 | 133 | 3178 | 3675 | 5183 | 2437 | 2457 | Gly-TCC | tRNA-5 |
| GTTTCCGTAGTGTAGTGGTTATCACGTTCGCC | 3.82 | 0.008 | 1706 | 1485 | 2562 | 2365 | 357 | 8409 | 11454 | 24313 | 12938 | 4006 | Val-AAC | tRNA-5 |
| GTCAGGATGGCCGAGCGGTCTAAGGCGCTGCG | 5.99 | 0.012 | 0 | 0 | 0 | 0 | 0 | 44 | 90 | 99 | 70 | 360 | Leu-CAG | tRNA-5 |
| GTTTCCGTAGTGTAGTGGTTATCACGTTCGC | 3.37 | 0.013 | 2043 | 1796 | 1708 | 2295 | 275 | 4961 | 8470 | 15770 | 7539 | 8559 | Val-AAC | tRNA-5 |
| GTTTCCGTAGTGTAGTGGTCATCACGTTCGCCT | 3.42 | 0.013 | 212 | 138 | 146 | 211 | 20 | 864 | 1215 | 1223 | 454 | 735 | Val-AAC | tRNA-5 |
| TTCCGTAGTGTAGTGGTTATCACGTTCGCC | 5.50 | 0.015 | 0 | 0 | 34 | 0 | 0 | 98 | 168 | 374 | 93 | 172 | Val-AAC | tRNA-i |
| TCCCTGGTGGTCTAGTGGTTAGGATTCGGCG | 3.10 | 0.022 | 7336 | 5111 | 3326 | 7471 | 1407 | 15378 | 25253 | 39671 | 25910 | 14129 | Glu-CTC | tRNA-5 |
| GGGGGATTAGCTCAAATGGT | -3.31 | 0.023 | 5019 | 2400 | 2202 | 375 | 133 | 117 | 72 | 93 | 273 | 156 | Ala-AGC | tRNA-5 |
| TATAGTGGTTAGTACTCTGCGTTGT | -4.99 | 0.023 | 50 | 173 | 202 | 164 | 112 | 0 | 0 | 0 | 6 | 31 | His-GTG | tRNA-i |
| ACGCAGAAGGTCCTGGGTTCGAGCCCCAGTGGAACCACCA | 4.03 | 0.024 | 62 | 17 | 11 | 0 | 31 | 102 | 481 | 434 | 128 | 172 | Val-TAC | tRNA-3 |
| GCATGGGTGGTTCAGTGG | -3.68 | 0.024 | 299 | 121 | 90 | 375 | 194 | 7 | 18 | 10 | 29 | 0 | Gly-GCC | tRNA-5 |
| GCTTCTGTAGTGTAGTGGTTATCACGTTCGCCT | 3.12 | 0.024 | 137 | 259 | 270 | 234 | 102 | 627 | 956 | 1763 | 937 | 595 | Val-CAC | tRNA-5 |
| AACCGGGCGGAAACACCA | -3.64 | 0.024 | 9802 | 11293 | 16999 | 61852 | 104908 | 1181 | 2990 | 714 | 16108 | 1565 | Val-AAC | tRNA-3 |
| CATTGGTGGTTCAGTGGTAGAATTCTCGC | 3.23 | 0.024 | 174 | 17 | 225 | 211 | 31 | 354 | 698 | 1049 | 471 | 1080 | Gly-CCC | tRNA-i |
| GCGCCGCTGGTGTAGTGGTATCATGCAAGA | 2.92 | 0.024 | 573 | 742 | 404 | 656 | 92 | 1177 | 1372 | 3194 | 2071 | 3098 | Gly-CCC | tRNA-5 |
| GGTGCGAGAGGTCCCGGGTTC | 5.14 | 0.024 | 0 | 0 | 45 | 0 | 0 | 22 | 529 | 106 | 17 | 673 | Pro-AGG | tRNA-i |
| GTCAGGATGGCCGAGCGGTCTAAGGCGCTGCGTT | 3.18 | 0.024 | 62 | 35 | 236 | 281 | 102 | 1177 | 824 | 1062 | 942 | 282 | Leu-CAG | tRNA-5 |
| GTCAGGATGGCCGAGCGGTCTAAGGCGCTGCGTTC | 2.94 | 0.024 | 361 | 345 | 562 | 328 | 71 | 656 | 1853 | 2150 | 942 | 1674 | Leu-CAG | tRNA-5 |
| TCCCACATGGTCTAGCGGTTAGGATTCCTGGTTT | 3.15 | 0.024 | 374 | 1312 | 337 | 468 | 102 | 1943 | 2527 | 4269 | 2600 | 876 | Glu-TTC | tRNA-5 |
| TCCCTGGTGGTCTAGTGGTTAGGATTCGGC | 2.76 | 0.024 | 2616 | 1416 | 1168 | 3677 | 958 | 5894 | 8698 | 11402 | 7405 | 9576 | Glu-CTC | tRNA-5 |
| TCCCACATGGTCTAGCGGTTAGGA | -2.75 | 0.030 | 1868 | 2469 | 4573 | 820 | 530 | 310 | 174 | 47 | 605 | 125 | Glu-TTC | tRNA-5 |
| AGCAGAGTGGCGCAGCGGAAGCGTGCTGGGCC | 3.02 | 0.031 | 87 | 35 | 34 | 141 | 71 | 222 | 457 | 506 | 419 | 532 | iMet-CAT | tRNA-5 |
| GTTTCCGTAGTGTAGTGGTCATCACGTTCGC | 4.04 | 0.031 | 12 | 0 | 22 | 70 | 0 | 36 | 211 | 244 | 134 | 376 | Val-AAC | tRNA-5 |
| GTTTCCGTAGTGTAGTGGTCATCACGTTCGCC | 4.33 | 0.036 | 0 | 0 | 112 | 0 | 20 | 157 | 367 | 636 | 244 | 31 | Val-AAC | tRNA-5 |
| GCGTTGGTGGTATAGTGGTTAGCATAGCTGC | 2.63 | 0.037 | 1407 | 345 | 438 | 703 | 184 | 3299 | 1582 | 2511 | 1943 | 3223 | Gly-TCC | tRNA-5 |
| CCCCACTCCTGGTACCA | -3.78 | 0.037 | 212 | 52 | 191 | 1148 | 1295 | 91 | 12 | 13 | 128 | 0 | Leu-TAA | tRNA-3 |
| GCTTCTGTAGTGTAGTGGTTATCACGTTCGC | 3.87 | 0.037 | 112 | 0 | 79 | 23 | 0 | 200 | 265 | 680 | 175 | 125 | Val-CAC | tRNA-5 |
| TCCCTGGTGGTCTAGTGGTTAGGATTCGGCGC | 2.44 | 0.039 | 16229 | 16645 | 13830 | 14520 | 6862 | 63685 | 43077 | 58183 | 41414 | 27694 | Glu-CTC | tRNA-5 |
| ATCTAAAGGTCCCTGGTTCGATCCCGGGTTTCGGCACCA | 3.63 | 0.042 | 0 | 35 | 56 | 23 | 0 | 456 | 78 | 49 | 250 | 266 | Phe-GAA | tRNA-3 |
| CACGCGGGAGACCGGGGTTCGATTCCCCGACGGGGAG | 2.94 | 0.042 | 336 | 207 | 225 | 234 | 20 | 3109 | 1263 | 434 | 448 | 266 | Asp-GTC | tRNA-i |
| GCGTTGGTGGTATAGTGGTTAGCATAGCTGCC | 2.72 | 0.043 | 187 | 328 | 236 | 141 | 71 | 445 | 969 | 1135 | 448 | 626 | Gly-TCC | tRNA-5 |
| AGCAGAGTGGCGCAGCGGAAGCGTGCTGGGC | 2.82 | 0.046 | 162 | 69 | 45 | 141 | 143 | 809 | 1005 | 587 | 471 | 172 | iMet-CAT | tRNA-5 |
| CGATTCCCGGCCAATGCAC | -4.59 | 0.046 | 648 | 155 | 124 | 70 | 0 | 0 | 0 | 3 | 29 | 0 | Gly-GCC | tRNA-i |
| GCGTTGGTGGTATAGTGGTGAGCATAGCTGCCTTC | 3.54 | 0.046 | 12 | 69 | 112 | 0 | 0 | 146 | 301 | 377 | 198 | 203 | Gly-TCC | tRNA-5 |
| GTTTCCGTAGTGTAGCGGTTATCACATTCGCC | 4.57 | 0.046 | 25 | 0 | 0 | 23 | 0 | 7 | 96 | 239 | 128 | 63 | Val-CAC | tRNA-5 |
| GTTTCCGTAGTGTAGTGGTTATCACGTTCGCCT | 2.51 | 0.046 | 7087 | 6855 | 6089 | 4661 | 1183 | 13552 | 19214 | 25349 | 13176 | 11469 | Val-AAC | tRNA-5 |
| TCCCGGGTTTCGGCACCA | -3.68 | 0.046 | 673 | 691 | 1607 | 8736 | 19740 | 237 | 265 | 78 | 3060 | 141 | Phe-GAA | tRNA-3 |
| CACGCAGAAGGTCCTGGGTTCGAGCCCCAGTGGAACCA | 4.29 | 0.047 | 0 | 35 | 0 | 0 | 10 | 470 | 96 | 44 | 47 | 63 | Val-TAC | tRNA-i |
| CATGGGTGGTTCAGTGGTAGAATTCTCGCCTG | 5.21 | 0.047 | 0 | 17 | 0 | 0 | 0 | 77 | 102 | 177 | 6 | 0 | Gly-GCC | tRNA-i |
| GATTCCCGGCCAATGCACCA | -3.48 | 0.047 | 498 | 311 | 876 | 703 | 153 | 0 | 12 | 0 | 128 | 94 | Gly-GCC | tRNA-3 |
| GCGTTGGTGGTATAGTGGTGAGCATAGCTGC | 2.38 | 0.047 | 473 | 656 | 270 | 515 | 173 | 1709 | 1137 | 1670 | 1297 | 1127 | Gly-TCC | tRNA-5 |
| TTCCCGGCCAATGCACCA | -3.35 | 0.047 | 1856 | 1260 | 2944 | 10539 | 20117 | 211 | 740 | 114 | 3874 | 235 | Gly-CCC | tRNA-3 |
| TTCCCGGGCGGCGCACCA | -3.40 | 0.047 | 1756 | 4023 | 5809 | 19415 | 51878 | 1312 | 999 | 390 | 8336 | 438 | Gly-CCC | tRNA-3 |
| TTTCCGTAGTGTAGTGGTTATCACGTTCGCC | 4.19 | 0.047 | 50 | 0 | 0 | 94 | 0 | 22 | 307 | 517 | 442 | 31 | Val-AAC | tRNA-i |
| GCGTTGGTGGTATAGTGGTGAGCATAGCTGCC | 2.95 | 0.048 | 187 | 35 | 146 | 94 | 10 | 459 | 680 | 558 | 227 | 141 | Gly-TCC | tRNA-5 |

Note: FC: fold change

**Table S3. List of differentially expressed tsRNA in patients for RT-qPCR**

| tsRNA-ID | Sequence |
| --- | --- |
| tRNA-GlyTCC-5 | GCGTTGGTGGTATAGTGGTGAGCATAGCTGCCTT |
| tRNA-ValAAC-5 | GTTTCCGTAGTGTAGTGGTTATCACGTTCGCC |
| tRNA-GluCTC-5 | TCCCTGGTGGTCTAGTGGTTAGGATTCGGCG |
| tRNA-ValTAC-3 | ACGCAGAAGGTCCTGGGTTCGAGCCCCAGTGGAACCACCA |

**Table S4. Primers for Reverse Transcription and Quantitative PCR**

| Primer name | Primer sequence |
| --- | --- |
| miR-16 RT-stem-loop | GTCGTATCCAGTGCAGGGTCCGAGGTATTCGCACTGGATACGACCGCCAATA |
| miR-16 qPCR forward primer | GTCGCCGTAGCAGCACGTAAA |
| tRNA-GlyGCC-5 T-stem-loop | GTCGTATCCAGTGCAGGGTCCGAGGTATTCGCACTGGATACGACGAGAAT |
| tRNA-GlyGCC-5 qPCR forward primer | GATGACGCATGGGTGGTTCAG |
| tRNA-ValAAC-5 RT-stem-loop | GTCGTATCCAGTGCAGGGTCCGAGGTATTCGCACTGGATACGACAGGCGAA |
| tRNA-ValAAC-5 qPCR forward primer | GAGGACGTTTCCGTAGTGTAGTGG |
| tRNA-ValTAC-5 RT-stem-loop | GTCGTATCCAGTGCAGGGTCCGAGGTATTCGCACTGGATACGACAGGCGAA |
| tRNA-ValTAC-5 qPCR forward primer | GGTGCTGACGGTTCCATAGTGTAGT |
| tRNA-GlyTCC-5 RT-stem-loop | GTCGTATCCAGTGCAGGGTCCGAGGTATTCGCACTGGATACGACAAGGCAGC |
| tRNA-GlyTCC-5 qPCR forward primer | GACGACGCGTTGGTGGTATAGTG |
| tRNA-ValAAC-5 RT-stem-loop | GTCGTATCCAGTGCAGGGTCCGAGGTATTCGCACTGGATACGACGGCGAACG |
| tRNA-ValAAC-5 qPCR forward primer | GACGACGTTTCCGTAGTGTAGTGGT |
| tRNA-GluCTC-5 RT-stem-loop | GTCGTATCCAGTGCAGGGTCCGAGGTATTCGCACTGGATACGACCGCCGAAT |
| tRNA-GluCTC-5 qPCR forward primer | GACAACTCCCTGGTGGTCTAGTG |
| tRNA-ValTAC-3 RT-stem-loop | GTCGTATCCAGTGCAGGGTCCGAGGTATTCGCACTGGATACGACTGGTGGTT |
| tRNA-ValTAC-3 qPCR forward primer | GACATCACGCAGAAGGTCCTGG |
| Universal qPCR reverse primer | CCAGTGCAGGGTCCGAGGTA |
